# 6.4 BRAIN FUNCTIONAL CONNECTOMICS BASED ON RESTING STATE FMRI: FROM NODES TO NETWORKS

**DOI:** 10.1093/schbul/sby014.021

**Published:** 2018-04-01

**Authors:** Juan Zhou

**Affiliations:** Duke-National University of Singapore Medical School

## Abstract

**Background:**

Emerging evidence suggests that psychosis arises from disrupted communication between distributed neural networks. In the past decade, network-sensitive neuroimaging methods have made it possible to examine vulnerable brain networks in living humans. Previous work has demonstrated that distinct functional intrinsic connectivity networks can be mapped in the healthy brain with task-free or “resting-state” functional magnetic resonance imaging (fMRI). Instead of the changes evoked by specific stimuli, resting state fMRI captures the spontaneous low frequency blood-oxygenation-level-dependent signal fluctuations at rest. Regions showing synchronized spontaneous activities are usually functionally connected and are often supporting highly relevant brain functions.

Being more applicable in patients, recent resting state fMRI studies in psychosis have reported widespread functional dysconnectivity, targeting multiple neural systems that include the default mode network, the salience network, the auditory network, and fronto-striato-thalamic circuits. Such functional connectivity disruptions are also associated with more severe symptoms and more cognitive impairments in patients.

**Methods:**

In this talk, I will cover four primary methods for deriving functional connectivity from resting state fMRI data and discuss their pros and cons in the context of schizophrenia. 1) Seed-based approach: correlation between signals of a seed region to other target regions or with the rest of the brain. 2) Independent component analysis: decompose the fMRI data of all brain voxels into spatially non-overlapping and temporally coherent networks. 3) Brain parcellation-based connectivity matrices: based on a set of predefined regions of interest covering the whole brain, the functional connectivity between all pairs of regions are computed and the individual-level connectivity matrices are compared. Lastly, 4) graph theoretical approach is highly useful in capturing and visualizing complex brain interactions embedded in these high dimensional matrices. In a brain graph, each ROI is a node and the functional connectivity between a pair of ROIs is an edge. Graph theoretical measures can then capture the brain functional topology such as functional segregation or modularity at nodal, network, and whole-brain levels.

**Results:**

By modelling connectivity as complex networks, this talk will shed some light on whether functional connectomics based on resting state fMRI could 1) reveal symptoms-associated brain network changes; 2) detect early changes in prodromal stage of the disease; 3) predict clinical outcomes in psychosis. Particularly, work from our group and others on persons at-risk for psychosis will be discussed. Moreover, accumulating evidence suggests the influence of vigilance, motion, and physiological noise on functional connectivity measures. I will provide some tips on how to minimize these confounds and increase the reliability and reproducibility of functional connectomics measures.

**Discussion:**

Resting state fMRI provides a novel network-sensitive, immediately repeatable, non-invasive tool to examine human functional connectome. Future directions such as dynamic or time-varying functional connectivity which captures neural dynamics at a finer time scale will be briefly discussed. Further developed and integrated with brain structural connectivity measures, brain network functional connectomics may help us better understand heterogeneity in psychosis, reveal disease mechanism, predict and track disease progression, and monitor treatment response.

